# Clinical features of NK/T-cell EBV-associated LPD manifested as gastrointestinal symptoms in patients with normal immunity: a case report and literature review

**DOI:** 10.1186/s12876-021-01718-4

**Published:** 2021-06-10

**Authors:** Si-Zhu Wang, Ying-Huan Dai, Jie Zhang, Fang-Gen Lu, La-Mei Yan, Shan Wu

**Affiliations:** 1 Department of Gastroenterology, The Second Xiangya Hospital, Changsha, 410011 Hunan China; 2 Research Center of Digestive Disease, Central South University, Changsha, 410011 Hunan China; 3 Department of Pathology, The Second Xiangya Hospital of Central South University, Changsha, 410000 Hunan China

**Keywords:** Epstein–Barr virus, NK/T-cell lymphoproliferative disease, Intestine, Diagnosis, Immunocompetent

## Abstract

**Background:**

Epstein–Barr virus (EBV)-associated NK/T-cell lymphoproliferative disorder (LPD) involving the gastrointestinal tract is rarely observed in individuals with normal immunity. The atypical clinical, colonoscopic manifestations often confuse clinicians, leading to misdiagnosis and delays in the treatment.

**Case presentation:**

Herein, we reported on a single case of a patient with gastrointestinal symptoms. Several colonoscopies showed multiple irregular ulcerations, while biopsies showed colitis with infiltration of neutrophils or lymphocytes. After 2 months follow-up, the patient was diagnosed with the extranodal NK/T-cell lymphoma, nasal type, and was treated with thalidomide. Later on, a second check was performed on his first pathological sample. Immunohistochemistry revealed EBV associated NK/T-cell LPD.

**Conclusions:**

Multiple, multiform, and segmental gastrointestinal ulcers should be an indication for EBV infection, regardless of the presence of fever, lymphadenopathy, and hepatosplenomegaly. If EBV-associated NK/T-cell LPD is considered, serum EBV-DNA should be measured, and the tissue obtained by biopsy should be carefully analyzed for a positive expression of the EBER marker.

## Background

EBV-associated NK/T-cell LPD is a new category adopted by the World Health Organization (WHO) in 2008. Its features include an excessive lymphoid proliferation of T or NK cells [1]. It is different from simple proliferative diseases (such as infection mononucleosis) and typical neoplastic diseases (such as NK/T cell lymphoma). The diagnosis is mainly based on pathological results. It usually occurs in children and young adults and is more likely to develop in immunocompromised patients [2]. Gastrointestinal involvement in patients with normal immunity is very rare and the manifestations are not typical [3, 4].

Currently, there is limited available literature on this matter that is mostly based on sporadic case reports, most of which were misdiagnosed [3, 5, 6]. This disease shows a fulminant clinical course with poor prognosis, and relying solely on mucosal biopsy for diagnosis is not sufficient. Previous studies reporting on this disease have mainly focused on its pathology rather than clinical features. Nonetheless, reducing the rate of misdiagnosis and performing a more accurate and early diagnosis still remain as challenging issues.

The purpose of this report is to present a case of EBV associated NK/T-cell LPD, which manifested with gastrointestinal symptoms and multiple irregular ulcerations.

## Case presentation

**Fig. 1:**
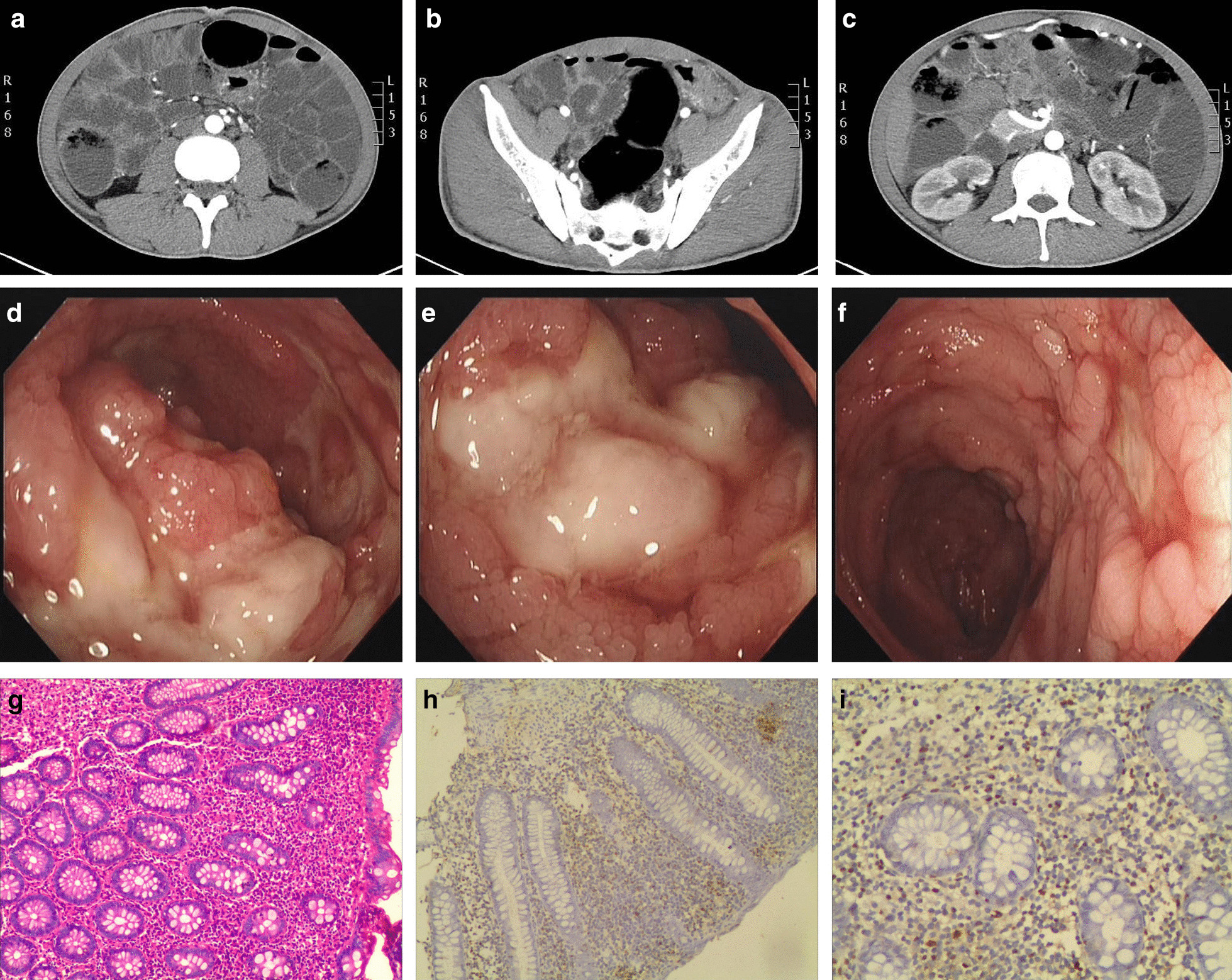
Wall thickness and enhancement after a contrast-enhanced scan in transverse colon (**a**), descending colon (**b**) and sigmoid (**c**). Segmental irregular and longitudinal ulcerations in the terminal ileum (**d**, **e**). Multiple irregular ulcerations in transverse colon (**f**). HE stain in × 100 magnification (**g**). EBER expression in × 100 magnification (**h**). EBER expression in × 200 magnification (**i**)

A 30-year-old Chinese man was admitted to the gastroenterology department at Second Xiangya Hospital. He complained of having intermittent fever, diarrhea for five years, and haematochezia for over 20 days (May 2016). The highest temperature was 41 °C. No obvious abnormal results were found during physical examination. The liver function test and renal function test were normal. Results of serologic tests for ENA, vasculitis, ANA, and ANCA were all negative. A T-SPOT test yielded negative results. GM test, G-test, HIV, TP, and serum virus laboratory tests (CMV-IgM, CMV-IgG, EBV-IgM, EBV-IgG) also showed negative results. Serum EBV-DNA was within the normal range. CT showed thickening of the transverse colon, descending colon, and the sigmoid wall (Fig. 1a–c). Several colonoscopies showed multiple irregular ulcerations in the terminal ileum and transverse colon, with segmental and longitudinal distribution (Fig. 1d–f). Several colonoscopic biopsies showed colitis with infiltration of neutrophils or lymphocytes in the lamina propria and formation of fissuring ulcers. The possibility of Crohn’s disease (CD) was considered. The patient was treated with antibiotics, ethyl-prednisolone, thalidomide and mesalazine. However, the symptoms were not relieved.

After 2 months, a neoplasm was found in the nasal cavity, after which a biopsy was performed. Histological examinations showed atypical lymphocyte hyperplasia with patchy necrosis and the destruction of some glandular structures. Immunohistochemistry result was CD3 (+), CD2 (++), CD20 (−), CD79α (−), CD56 (+), TiA-1 (+), CD21 (−), mum-1 (+), CD5 (−), CyclinD1 (−), TdT (−) and Ki-67 (80%+). The EBER in situ hybridization was > 100/HPF. The patient was diagnosed as extranodal NK/T-cell lymphoma, nasal type. Considering that his family members rejected chemotherapy, he was treated with thalidomide (50 mg Qn). The patient was followed for two years until 2018 and, currently (July 07, 2020), is still alive.

After he was diagnosed as extranodal NK/T-cell lymphoma, nasal type, we took in consideration the possibility of misdiagnosis. The first pathologic result was rechecked. Formation of multiple ulcers was discovered, accompanied by lymphocytes and plasmocytes infiltration in the mucous. Reduced glands, lymphocytic infiltration, and atypical lymphocytes were also observed. Immunohistochemistry result was CK (epithelium+), CD3 (10%+), CD4 (40%), CD20 (10%+), CD21 (−), Ki-67 (30%+), EBER in situ hybridization suggested 40/HPF (Fig. 1g–i). Therefore, the patient was suffering from EBV associated NK/T-cell LPD in the beginning.

## Discussion and conclusions

Herein, we reported a single case of a patient with EBV + NK/T-cell LPD who manifested chronic gastrointestinal symptoms. No sign of lymphoproliferative disorder was found in repeated colonoscopies. Therefore, the patient was treated for CD. Consequently, a neoplasm in the nasal cavity was found, and the patient was diagnosed with lymphoma. However, after the second analysis of a biopsy, the initial diagnosis was corrected to EBV + NK/T-cell LPD.

EBV + LPD is usually observed in patients with immune deficiencies [7]. However, some rare cases may manifest gastrointestinal symptoms [3]. The intestinal LPDs are the most frequent form of extranodal LPD with clinical presentation that is similar to that of IBD [8]. Therefore, in most cases misdiagnosis is commonly identified only after being mistreated or their condition deteriorates.

In the present study, we searched PubMed and CNKI for relevant literature published until May 2019 using the following keywords: ‘lymphoproliferative disease’ and ‘intestine’ and ‘EBV’. Only studies or case reports involving patients over 12 years old with normal immunity and T/NK cell lineage derived were found. Their initial symptoms were gastrointestinal symptoms, and the immunity was normal. In total, 24 patients were reported whose clinical features are summarized in Table 1 [3–7, 9–13].

**Table 1:** Clinical features of NK/T-cell EBV-associated LPD manifested as gastrointestinal symptoms

References	Country	Number of case	Age/sex	DCBC	Initial symptoms	Treatment history	Affected sites	Complications	Misdiagnosis	Endoscopic findings	Confirmation approach	Treatment
Shen et al. [4]	China	1	26/M	1 year	Diarrhea, fever	Ant anti-TB therapy	Colon	–	TB	Superficial ulcer, erythema, erosion	Lymph node biopsy	GLIDE
Shen et al. [4]	China	2	21/M	1 year	Diarrhea, fever	Antibiotics	NS	–	Intestinal infection	NS	Lymph node biopsy	Sodium phosphonate, interferon, prednisone
Chen et al. [13]	China	3	29/M	1 year	Diarrhea, abdominal pain	Methylprednisolone and mesalazine, methyl-prednisolone, anti-TNF treatment	Esophagus, stomach, IC and the entire colon	Perforation	CD	Multiple ulcers	Surgery	CHOP
Na et al. [3]	Korea	4	50/M	8 years	Loose stools	Antituberculous medication	IC,SC and rectum, jejunum	Perforation	TB	Multiple multiform ulcers	Endoscopy	CHOP
Na et al. [3]	Korea	5	49/F	19 months	Hematochezia	Prednisolone, azathioprine	IC, AC	Perforation	CD	Multiple well-demarcated circumferential or geographic ulcer	Endoscopy	DHAP
Zhu and Tang [14]	China	6	47/M	9 months	Diarrhea, fever	Anti-TB therapy	IC	–	TB	Multiple circumferential or geographic ulcer	Endoscopy	Thalidomide, Anflon, prednisone
Xiao et al. [15]	China	7	14/M	9 months	Abdominal pain, diarrhea, intermittent fever, hematochezia	Mesalazine and prednisone	IC, AC	Perforation	IBD	Ulcers and erosions	Surgery	Surgery
Furuya et al. [16]	China	8	49/F	44 months	Diarrhea and abdominal pain	5-Aminosalicylate	IC	–	CD	Longitudinal ulcers	Endoscopy	SMILE
Abdul-Ghafar et al. [7]	Korea	9	45/M	45 days	Diarrhea, weight loss	metronidazole	IC, AC, TC, DC, SC, rectum	Hematochezia	Infectious colitis	Multiple, variable sized, irregular shallow ulcerations	Endoscopy	–
Wang et al. [5]	China	10	54/M	5 years	Abdominal pain, haematochezia, fever	5-ASA, GC	AC, TC, DC, SC	Perforation, haemorrhage	UC, CD	MIVUs including a longitudinal ulcer	Surgery	–
Wang et al. [5]	China	11	31/F	10 years	Abdominal pain, haematochezia, fever	5-ASA, GC	Colorectum	Haemorrhage and HLH	UC	Diffuse inflammation, sporadic small ulcers	Surgery	GLIDE,HSCT
Wang et al. [5]	China	12	24/F	3 months	Haematochezia, fever	Anti-TB therapy	IC, AC, SC, rectum	–	TB	MIVUs	Endoscopy	GLIDE
Wang et al. [5]	China	13	49 M	1 year	Abdominal pain, weight loss	5-ASA, IFX [four times]	IC	Perforation	CD	Isolated annular ulcer	Endoscopy	–
Wang et al. [5]	China	14	40/M	3 years	Abdominal pain, haematochezia, fever	5-ASA	IC and whole colorectum	–	UC	Diffuse inflammation, sporadic ulcers	Endoscopy	GLIDE
Wang et al. [5]	China	15	35/M	3 years	Abdominal pain, haematochezia, fever	–	AC, TC, SC	Perforation	CD	Multiple irregular ulcers	Surgery	–
Wang et al. [5]	China	16	47/M	9 months	Diarrhea, fever	Anti-TB therapy	IC, AC, TC, DC, SC, rectum	–	TB	MIVUs including one transverse ulcer	Endoscopy	GLIDE
Wang et al. [5]	China	17	30/F	2 years	Abdominal pain, diarrhea, fever	5-ASA, Anti-TB therapy	TC, rectum	–	UC, CD	Two irregular [both transverse-like] ulcers	Endoscopy	GLIDE
Wang et al. [5]	China	18	39/F	4 years	Haematochezia, fever	–	IC, AC, TC, DC	Perforation	–	MIVUs including one transverse ulcer	Endoscopy	LVD
Wang et al. [5]	China	19	53/F	9 months	Abdominal pain, diarrhea, fever	5-ASA, GC	IC, AC, TC, DC, SC	–	CD	MIVUs including one transverse ulcer	Endoscopy	–
Wang et al. [5]	China	20	34/F	5 months	Abdominal pain, haematochezia, fever	–	Ileum	Perforation, obstruction	CD	Multiple round ulcers	Surgery	–
Wang et al. [5]	China	21	29/M	3 months	Abdominal pain, haematochezia, fever	Anti-TB therapy	IC, AC, DC, SC	Perforation	TB	MIVUs including one transverse ulcer	Endoscopy	–
Sazuka et al. [17]	Japan	22	71/F	3 months	Diarrhea	–	Small intestine	–	–	Flattened villi	Endoscopy	CHOP
Wang et al. [6]	China	23	26/M	1 year	Diarrhea and abdominal pain	IFX [five times]	IC, AC, TC, DC, SC	–	CD	Multiple nodular hyperplasia, deep ulcers	Endoscopy	CHOP
Our case	China	24	30/M	5 years	Fever and diarrhea	Ethyl-prednisolone, thalidomide and mesalazine	IC, TC	–	CD	MIVUs	Endoscopy	Thalidomide

Patients with EBV + T/NK-LPD and gastrointestinal symptoms, were mainly younger male individuals, which is consistent with the characteristics found in our case study. In addition, all patients were Asians, which suggests genetic susceptibility [2]. The most common clinical manifestation in patients with EBV + T/NK-LPD was fever (the temperature ranged from 37 to 41 °C) [1]. However, not all the patients had a fever; some patients developed fever after applying hormonal or anti-TNF [6]. Besides fever, diarrhea, abdominal pain, and hematochezia were also observed. In patients with chronic active EBV infective enteritis, diarrhea and abdominal pain were the most common gastrointestinal symptoms, along with fever, which was intermittent and mostly over 39 °C [17]. Briefly, the clinical manifestations of EBV + T/NK LPD, which manifested as gastrointestinal symptoms are atypical. Therefore, most patients were misdiagnosed with CD, infection, and so on [3, 6, 7].

As for laboratory inspection, the most common blood routine results were mild leukocytosis and anemia [4, 9–11]. Anemia was common in EBV + T/NK LPD [1], but it was still not specific due to its similarity to a CD. Liver function showed hypoalbuminemia [3, 4, 9–11], while transaminase elevation was rarely observed [3]. Bilirubin and coagulation function were also normal in the majority of EBV + T/NK LPD cases. However, liver dysfunction was observed in 77% EBV + T/NK LPD patients [11]; which may be related to the different initial infiltrating positions. Deep lymphadenopathy in CT was also commonly reported [3, 4, 6, 10, 11]; however, superficial lymphadenopathy and hepatosplenomegaly were rare [4]. According to Kimura et al. [1], hepatomegaly and lymphadenopathy are common in patients with EBV-associated T/NK-lymphoproliferative cell diseases. These symptoms are also commonly found in chronic active Epstein–Barr virus infective enteritis [17]. Briefly, deep lymphadenopathy may provide some general clue, which led to conclusion that lymphadenopathy and hepatosplenomegaly may happen in the late stage of the disease.

In terms of colonoscopy examination, multiple and multi-shaped ulcers, which were commonly found, were distributed in the gastrointestinal tract [5–7, 9]. No obvious segmental feature was noticed. Ulcers vary in shape, depth, and size [3, 5, 7]. Similar ulcers were also observed in patients with chronic active EBV infective enteritis [17]. Those ulcers lack of typical features of CD(Crohn’s disease) or ITB(Intestinal tuberculosis), like longitudinal or transverse ulcers, and cobblestone appearance [18]. Yet, there is still some other non-typical behavior in colonoscopies, such as diffusely atrophic small-intestinal villi and erythema [4, 12]. As for CT examination, segmental wall thickening was common [6, 11], which was similar to CD and ITB.

EBV has an etiological role in various diseases, including infectious mononucleosis, chronic active EBV infection, and malignancies such as nasopharyngeal cancer and Burkitt's lymphoma [19]. Serological studies of EBV infection include EBV antibody and EBV-DNA. Because the EBV infects > 90% of humans and persists during their lifetime, most people develop an EBV antibody [19]. Nevertheless, some patients may lack serum EBV-DNA duplication but be positive for EBER. In our case, the patient was negative for EBV-DNA but positive for EBER. EBER in situ hybridization suggested the presence of EBV-infected cells in the tissues. We assumed that EBV occurred only in intestinal tissue at an early stage of the disease. The detection of EBER seems to be more significant. EBER showing over 50/HP is considered meaningful generally. However, we found that some results didn’t conform to it, which may be related to the uneven EBV distribution in biopsy specimens. This in turn suggests that multiple and multifocal specimens are necessary when considering the possibility of EBV + disease.

Considering the indistinguishable clinical manifestations from other intestinal disorders, EBV + NK/T-LPD is diagnosed based on pathology. Some patients developed lymphoma. A typical lymphocytic proliferation with varied sized lymphocytes is also important. In patients with EBV + NK/T-LPD, lymphocytes may infiltrate other sites, such as lymph node and vessel. As for immunohistochemistry, CD3, CD56, TIA-1, and EBER are commonly expressed; Ki-67 ranges from 5 to 80% [4–6, 11]. In our cases, at the early stage of the disease, Ki-67 was 30% and then increased to 80%, which indicated that Ki-67 might be associated with the disease progression. Moreover, Wang et al. [5] analyzed the pathological features of the biopsies in the early stage of EBV + NK/T-LPD and found that these tissues were characterized by small- or medium-sized lymphoid cells, ALCs, LELs, and angiocentric infiltrations. EBER-positive cells can be detected by in situ hybridizations.

According to Kimura et al., in a median follow-up period of 46 months, almost half of EBV + NK/T-LPD died of severe organ complications. We found that some cases developed lymphoma [1, 5, 6]. Currently, there is still no standard treatment for EBV + NK/T-LPD. Patients with post-transplant lymphoproliferative disorders are usually treated with RIS (reduction of immune suppression) and other treatment modalities (chemotherapy, radiotherapy, or surgery). Yet, the response is still poor [20]. Sakai et al. has shown that interferon has a good effect on patients with chronic active Epstein–Barr virus infection [21]. Thalidomide is a potential therapeutic strategy for ENKTL-associated HPS [22]. In clinic, the main treatments include interferon, thalidomide to chemotherapy, and HSCT [3, 5, 6]. However, the overall death rate is still high. Therefore, new treatment modalities should be investigated.

EBV-associated NK/T LPD accompanied by gastrointestinal symptoms is rare in patients with normal immunity. These patients generally have a poor prognosis and a median survival of only a few months despite intensive chemotherapy [23]. Multiple, multiform, and segmental gastrointestinal ulcers should be an indication for EBV infection, regardless of the presence of fever, lymphadenopathy, and hepatosplenomegaly. If Epstein–Barr virus-associated NK/T-cell LPD is considered, serum EBV-DNA should be measured, and the tissue obtained by biopsy should be carefully analyzed for a positive expression of EBER marker.

## Data Availability

All data generated or analysed during this study are included in this published article.

## Abbreviations

EBV: Epstein–Barr virus
LPD: Lymphoproliferative disorder
WHO: World Health Organization
CD: Crohn’s disease
